# Association between sidedness and survival among chemotherapy refractory metastatic colorectal cancer patients treated with trifluridine/tipiracil or regorafenib

**DOI:** 10.1093/oncolo/oyae235

**Published:** 2024-09-07

**Authors:** Kai-Yuan Hsiao, Hsin-Pao Chen, Kun-Ming Rau, Kuang-Wen Liu, Ben-Chang Shia, Wei-Shan Chang, Hao-Yun Liang, Meng-Che Hsieh

**Affiliations:** Artificial Intelligence Development Center, Fu Jen Catholic University, New Taipei City, Taiwan; Graduate Institute of Business Administration, College of Management, Fu Jen Catholic University, New Taipei City, Taiwan; Division of Colon and Rectum Surgery, Department of Surgery, E-Da Hospital, Kaohsiung, Taiwan; College of Medicine, I-Shou University, Kaohsiung, Taiwan; College of Medicine, I-Shou University, Kaohsiung, Taiwan; Department of Hematology-Oncology, E-Da Cancer Hospital, Kaohsiung, Taiwan; Division of Colon and Rectum Surgery, Department of Surgery, E-Da Hospital, Kaohsiung, Taiwan; College of Medicine, I-Shou University, Kaohsiung, Taiwan; Artificial Intelligence Development Center, Fu Jen Catholic University, New Taipei City, Taiwan; Graduate Institute of Business Administration, College of Management, Fu Jen Catholic University, New Taipei City, Taiwan; Artificial Intelligence Development Center, Fu Jen Catholic University, New Taipei City, Taiwan; Graduate Institute of Business Administration, College of Management, Fu Jen Catholic University, New Taipei City, Taiwan; College of Medicine, I-Shou University, Kaohsiung, Taiwan; EMMT Systems Corporation, Director, Taiwan; College of Medicine, I-Shou University, Kaohsiung, Taiwan; Department of Hematology-Oncology, E-Da Cancer Hospital, Kaohsiung, Taiwan

**Keywords:** sidedness, metastatic colorectal cancer, trifluridine/tipiracil, TAS-102, regorafenib, sequence

## Abstract

**Background:**

The impact of sidedness on survival of later-line treatment in patients with metastatic colorectal cancer (mCRC) is undetermined. This study aimed to investigate the association between sidedness and survival among chemotherapy refractory patients with mCRC treated with trifluridine/tipiracil (TAS-102) or regorafenib or both.

**Patients and Methods:**

Patients with mCRC treated with TAS-102 or regorafenib between 2015 and 2020 was retrospectively collected. Patients were stratified into TAS-102 first and regorafenib first, then subdivided into TAS-102 followed by regorafenib (T-R) and regorafenib followed by TAS-102 (R-T) groups. The oncologic outcomes were presented with time-to-treatment failure (TTF) and overall survival (OS).

**Results:**

After matching, 376 TAS-102 patients and 376 regorafenib patients were included for outcomes comparison. TTF had insignificant differences while OS was significantly different between TAS-102 and regorafenib groups. Median TTF and OS were 1.9 months versus 2.0 months (*P* = .701) and 9.1 months versus 7.0 months (*P* = .008) in TAS-102 and regorafenib, respectively. The OS benefits were consistent regardless primary tumor location. Subgroup analysis with 174 T-R patients and 174 R-T patients was investigated for treatment sequences. TTF and OS had significant differences in both groups. Median TTF and OS were 8.5 months versus 6.3 months (*P* = .001) and 14.4 months versus 12.6 months (*P* = .035) in T-R and R-T groups, respectively. The TTF and OS benefits were persisted regardless primary tumor location.

**Conclusion:**

TAS-102 first provided a better survival benefit in chemotherapy refractory patients with mCRC across all sidedness. Further prospective studies are warranted to validate our conclusions.

## Teaser

This study investigated the association between sidedness and survival among patients with chemotherapy refractory metastatic colorectal cancer treated with trifluridine/tipiracil (TAS-102) or regorafenib or both.

**Implications for practice**

This study investigated the association between sidedness and survival in the largest reported cohort of patients.

## Introduction

Colorectal cancer (CRC) is one of the most common gastrointestinal tract cancers and the third prevalent malignancy in the world, with more than 1.9 million new patients with CRC and 935 000 deaths owing to CRC in 2020.^1^ It is also the second most common cause of cancer-related death in the US.^2^ In 2023, there were an estimated 153 020 new cases and 52 550-related deaths in the US.^3^ Approximately 20% of patients with CRC has metastatic disease at diagnosis^4,5^ and more than half patients develop metastases over their treatment course eventually.^6^ Metastatic CRC (mCRC) is associated with a poor prognosis, with a 5-year survival rate of about 14%.^2^ The standard treatment for patients with unresectable or metastatic CRC (mCRC) includes chemotherapy based on fluoropyrimidines, oxaliplatin, and irinotecan^7^ combined with anti-vascular endothelial growth factor (VEGF) antibodies^8,9^ and anti-epidermal growth factor receptor (EGFR) antibodies^10-12^ in the case of RAS wild-type tumors. Remarkable progress in the development of efficacious drugs for metastatic colorectal cancer (mCRC) has prolonged the median overall survival (OS) from first-line therapy up to 30 months.^13^ However, mCRC is still one of the major causes of cancer-related death, and many patients experience a phase that is refractory to conventional cytotoxic and molecularly targeted agents.^14^ Although these drugs have extended the median OS of patients with mCRC,^15-17^ these outcomes are not satisfactory and the developments of new active drugs for chemotherapy refractory mCRC or investigations of optimal treatment sequence are needed.

For chemotherapy refractory mCRC, 2 approved oral agents, trifluridine/tipiracil (TAS-102; Taiho Pharmaceutical, Tokyo, Japan; TTY Pharmaceutical, Taipei, Taiwan)^18^ and regorafenib (Stivarga; Bayer AG, Berlin, Germany),^19^ have been proved to prolong survival in randomized, multicenter phase III clinical trials. Both agents are indicated for the treatment of patients with mCRC who have been previously treated with fluoropyrimidine-, oxaliplatin-, and irinotecan-based chemotherapy or anti-vascular endothelial growth factor therapy and anti-epidermal growth factor receptor therapy (if RAS wild type). Previous studies had compared oncologic outcomes between regorafenib and TAS-102 treatments in chemotherapy refractory patients with mCRC.^20-22^ However, the impact of sidedness on survival of later-line treatment in patients with mCRC is undetermined. Thus, this study aimed to investigate the association between sidedness and survival among chemotherapy refractory patients with mCRC treated with TAS-102 or regorafenib or both.

## Methods

### Data source

The National Health Insurance Research Database (NHIRD) covers over 99% of the population in Taiwan. Taiwan Cancer Registry (TCR) had >98% overall coverage of potential cancer patients in Taiwan.^23^ The cancer diagnosis in TCR was confirmed by histological or cytological exams. This retrospective nationwide population-based study was conducted using the data from NHIRD and TCR, which comprised the information on cancer diagnosis, staging, and treatment. For privacy and data security, all personal identification numbers in the NHIRD have been concealed by the National Health Research Institute. In addition, diagnostic and procedural codes were classified according to the International Classification of Diseases, 9th revision, clinical modification (ICD-9-CM) coding system. This study was approved by the Institutional Review Board of the E-Da Cancer Hospital in Taiwan (EMRP-110-167), and the requirements for informed consent were waived.

### Study population

In this population-based study, we retrospectively identified patients who aged 18 years and older with newly diagnosed mCRC (ICD-9-CM code 153-154, ICD-10-CM code C18, C19, C20) between 2015 and 2020 from TCR. The Taiwan’s National Health Insurance Administration (NHIA) checks the accuracy of diagnosis regularly from electronic medical records that includes adjudication and profile analysis. Moreover, a small percentage of patients is selected for professional review by physicians with various specialties for the rationality of medical procedures and services. The definition of chemotherapy refractory patients with mCRC are those after oxaliplatin, irinotecan, 5-fluorouracil, anti-VEGF antibody, and anti-EGFR antibody if ras wild type. Chemotherapy refractory patients with mCRC who were treated with either TAS-102 or regorafenib were included into our study. Patients with treatment duration <1 month and no clear sidedness of colon cancer were excluded from our study. Patients were stratified into TAS-102 first and regorafenib first according to their treatment, then subdivided into TAS-102 followed by regorafenib (T-R) and regorafenib followed by TAS-102 (R-T) groups according to their treatment sequences. Propensity score matching was used to diminish selection bias with age, gender, hypertension, diabetic mellitus, chronic kidney disease, and year of treatment as matching factors.

### Covariates

The covariates in our analysis included patient’s age, gender, primary tumor location, initial stage, previous surgery, ras status, and comorbidities. The sidedness of colon cancer was classified into 2 part: left-side colon, including descending colon, sigmoid colon, rectum (ICD-9-CM 153.0-153.3, 154, ICD-10-CM C18.5-C18.7, C19, C20), and right-side colon, including ascending colon and transverse colon (ICD-9-CM 153.4-153.7, ICD-10-CM C18.0-C18.4). Patients with uncertain primary tumor location (ICD-9-CM 153.8-153.9, ICD-10-CM C18.8-C18.9) were excluded from our study. The following comorbidities were selected: diabetes (ICD-9-CM codes 250), hypertension (401–405), chronic kidney disease (585–586), and chronic hepatitis (571–573) which were diagnosed before the index date. The chemotherapy was selected based on the Anatomical Therapeutic Chemical (ATC) system of medications of L01BC59, L01EX05 from the NHIRD, respectively.

### Statistical analysis

All characteristics were all presented with frequencies. Differences in the distribution of characteristics were examined using chi-squared tests. In order to reduce the selection bias, patients with TAS-102 treatment were matched with patients with regorafenib by using propensity score matching according to age, gender, sidedness, initial stage, and comorbidities. The oncologic outcomes were presented with time-to-treatment failure (TTF) and OS. The index date was defined as the date of firstly starting TAS-102 or regorafenib. In primary analysis, TTF was defined as the time from the index date to death or the last date of treatment while OS was defined as the time from the index date to death or the last visiting. In subgroup analysis of treatment sequence, TTF was defined as the time from the index date to death or the last date of TAS-102 and regorafenib while OS was defined as the time from the index date to death or the last visiting. Kaplan–Meier curves were estimated for TTF and OS. Surviving patients were censored at their time of last follow-up. We conducted a log-rank test with Cox regression models to adjust for the effects of potential confounders. Hazard ratios (HR) and 95% confidence intervals (CI) were estimated. Furthermore, we further evaluate the association between sidedness and survival among patients treated with TAS-102 or regorafenib. Kaplan–Meier curves and Cox regression analysis were repeated with sidedness. All *P* values were two sided and considered to be statistically significant if *P* values < .05.

## Results

**Figure 1.**
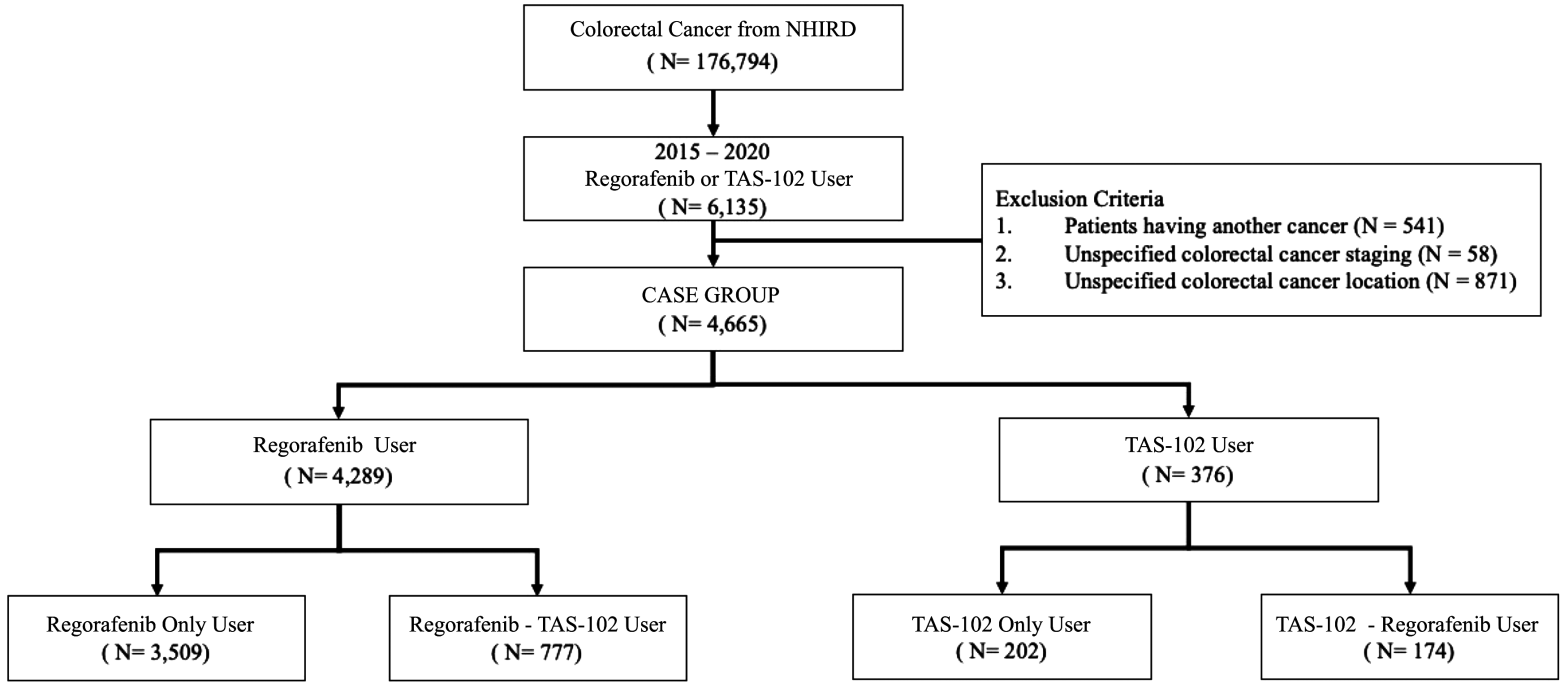
A flow diagram of patient selection and exclusion.

**Figure 2.**
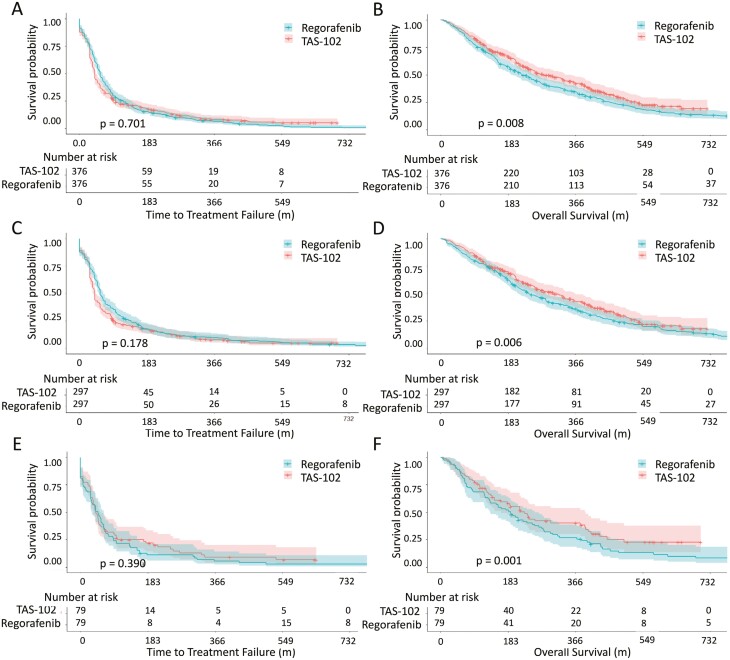
TTF and OS of metastatic colorectal cancer treated with TAS-102 or regorafenib. A and B, TTF and OS of total metastatic colorectal cancer treated with TAS-102 or regorafenib. C and D, TTF and OS of metastatic left-side colorectal cancer treated with TAS-102 or regorafenib. E and F, TTF and OS of metastatic right-side colorectal cancer treated with TAS-102 or regorafenib.

We identified patients with mCRC treated with either TAS-102 or regorafenib between 2015 and 2020 from NHIRD and TCR (Figure 1). Among the 4665 eligible patients, 376 patients were in TAS-102 group and 4289 patients were in regorafenib group. Table 1 shows the distributions of characteristics between TAS-102 and regorafenib groups. The median age was older in TAS-102 as compared with those in regorafenib. Male was the predominant gender in both groups. In terms of sidedness, 80% had left side colon cancer and approximately 20% had right-side colon cancer regardless TAS-102 group or regorafenib. More than 80% of our patients was diagnosed to have clinical stages III and IV in both groups. Nearly 60% of patients had previous history of radical colectomy in both groups. As for all RAS status, 55% had RAS mutant while 45% had RAS wild type in our cohort. Among these RAS mutant patients with mCRC, 16% of our patients had KRAS G12 mutations, 7% had KRAS G13 mutations, and 14% had other KRAS mutations. There were 5% of our patients harboring BRAF mutant and 8% harboring dMMR (mismatch repair gene deficiency). With regard to comorbidities, more patients with diabetes and hypertension were found in TAS-102 group while more patients had chronic kidney disease were found in regorafenib group. After matching, 752 patients were analyzed for outcomes comparison, including 376 patients in TAS-102 group and 376 patients in regorafenib group. The median ages were 63.22 years and male were the predominant gender (55%) in both groups. There were no significant differences in age, gender, sidedness, initial stage, all RAS status, BRAF status, MMR status, previous history of colectomy, diabetes, hypertension nor chronic kidney disease between TAS-102 and regorafenib groups. The distribution of KRAS G12, G13, and other mutations was also balanced between TAS-102 and regorafenib arms, accounting for 16.6%, 6.3%, 14.4% in TAS-102 and 15.4%, 7.7%, 13.6% in regorafenib (*P* = .752). At the end of analysis, 68% of TAS-102 patients had subsequent treatments with 46% regorafenib and 22% chemotherapy re-challenge, while 52% of regorafenib patients had subsequent treatments with 40% TAS-102 and 12% chemotherapy re-challenge. Kaplan-Meier curves with TTF and OS were plotted for total population (Figure 2A and B). In total population, TTF had insignificant differences while OS were significantly different between TAS-102 and regorafenib groups. Median TTF and OS were 1.9 months versus 2.0 months (HR: 1.029, 95% CI: 0.887-1.193, *P* = .701) and 9.1 months versus 7.0 months (HR: 0.805, 95% CI: 0.678-0.957, *P* = .008) in TAS-102 and regorafenib, respectively.

**Table 1.** Basic characteristics of chemotherapy refractory patients with mCRC, stratified by PSM.

	PSM unmatched			PSM matched		
	TAS-102	Regorafenib	*P*	TAS-102	Regorafenib	*P*
*N*	376	4289		376	376	
Age (mean (SD))	63.22 (10.69)	61.59 (11.31)	.007	63.22 (10.69)	63.22 (10.69)	1.000
Gender						
Male	207 (55.1)	2490 (58.1)	.282	207 (55.1)	207 (55.1)	1.000
Female	169 (44.9)	1799 (41.9)		169 (44.9)	169 (44.9)	
Sideness						
Left	297 (79.0)	3292 (76.8)	.356	297 (79.0)	280 (74.5)	.167
Right	79 (21.0)	997 (23.2)		79 (21.0)	96 (25.5)	
Initial T stage						
T1-T2	57 (15.2)	575 (13.4)	.382	57 (15.2)	49 (13.0)	.463
T3-T4	319 (84.8)	3714 (86.6)		319 (84.8)	327 (87.0)	
Initial N stage						
N0-N1	220 (58.5)	2595 (60.5)	.482	220 (58.5)	227 (60.4)	.656
N2	156 (41.5)	1694 (39.5)		156 (41.5)	149 (39.6)	
Initial M stage						
M0	178 (47.3)	1950 (45.5)	.518	178 (47.3)	169 (44.9)	.558
M1	198 (52.7)	2339 (54.5)		198 (52.7)	207 (55.1)	
Initial stage						
I-II	69 (18.4)	691 (16.1)	.291	69 (18.4)	65 (17.3)	.775
III-IV	307 (81.6)	3598 (83.9)		307 (81.6)	311 (82.7)	
Previous colectomy						
No	148 (39.4)	1753 (40.9)	.605	148 (39.4)	170 (45.2)	.121
Yes	228 (60.6)	2536 (59.1)		228 (60.6)	206 (54.8)	
All RAS status						
Mutant	208 (55.3)	2313 (53.9)	.642	208 (55.3)	210 (55.9)	.941
Wild	168 (44.7)	1976 (46.1)		168 (44.7)	166 (44.1)	
BRAF status			.952			1.000
Mutant	20 (5.3)	218 (5.0)		20 (5.3)	20 (5.3)	
Wild	356 (94.7)	4071 (95.0)		356 (94.7)	356 (94.7)	
MMR status			.827			1.000
Proficiency	347 (92.3)	3925 (91.5)		347 (92.3)	347 (92.3)	
Deficiency	29 (7.7)	364 (8.5)		29 (7.7)	29 (7.7)	
Hypertension						
No	166 (44.1)	2006 (46.8)	.356	166 (44.1)	151 (40.2)	.301
Yes	210 (55.9)	2283 (53.2)		210 (55.9)	225 (59.8)	
Diabetic mellitus						
No	245 (65.2)	2907 (67.8)	.326	245 (65.2)	245 (65.2)	1.000
Yes	131 (34.8)	1382 (32.2)		131 (34.8)	131 (34.8)	
Chronic kidney disease						
No	344 (91.5)	3795 (88.5)	.092	344 (91.5)	335 (89.1)	.324
Yes	32 (8.5)	494 (11.5)		32 (8.5)	41 (10.9)	

Abbreviations: MMR, mismatch repair gene; PSM, propensity score match.

Patients were stratified according to sidedness, accounting for 594 patients with left-side colon cancer and 158 patients with right-side colon. For patients with left-side colon cancer, 297 patients treated with TAS-102 and 297 patients treated with regorafenib. For patients with right side colon cancer, 79 patients treated with TAS-102 and 79 patients treated with regorafenib. Table 2 summarized the characteristics of patients in each group. The median age was 63 years in patients with left-side colon and the median age was 62 years in patients with right-side colon. There were no significant differences in age, gender, sidedness, initial stage, all RAS status, BRAF status, MMR status, previous history of colectomy, diabetes, hypertension nor chronic kidney disease between TAS-102 and regorafenib groups no matter for patients with left-side colon cancer or right-side colon cancer. Figure 2C and D plotted the TTF and OS of left-side colon group with Kaplan-Meier curves. For patients with left side colon, median TTF and OS were 1.9 months versus 2.0 months (HR: 1.158, 95% CI: 0.979-1.370, *P* = .178) and 10.2 months versus 7.8 months (HR: 0.803, 95% CI: 0.658-0.981, *P* = .006) in TAS-102 and regorafenib, respectively. Figure 2E and F plotted the TTF and OS of right-side colon group with Kaplan-Meier curves. For patients with right-side colon, median TTF and OS were 1.8 months versus 1.8 months (HR: 0.977, 95% CI: 0.664-1.322, *P* = .390) and 8.6 months versus 6.0 months (HR: 0.744, 95% CI: 0.518-0.907, *P* = .001) in TAS-102 and regorafenib, respectively. Table 3 shows Cox regression analyses of OS in total population with hazard ratio (HR) and 95% CIs (confidence intervals). In univariate analysis, age, initial stage, all-ras status, previous history of colectomy, diabetes, hypertension nor chronic kidney disease were insignificant with survival. In contrary, treatment with TAS-102, male, left-side colon cancer, initial M0 disease was independent prognosticators with OS. After adjusting potential confounding factors with multivariate analysis, treatment with TAS-102, male, left-side colon cancer remained significant correlated with OS.

**Table 2.** Basic characteristics of chemotherapy refractory patients with mCRC, stratified by sideness.

	Left side			Right side		
	TAS-102	Regorafenib	*P*	TAS-102	Regorafenib	*P*
N	297	297		79	79	
Age (mean(SD))	63.57 (10.86)	63.57 (10.86)	1.000	61.89 (9.98)	62.32 (10.11)	.788
Gender						
Male	175 (58.9)	175 (58.9)	1.000	32 (40.5)	31 (39.2)	1.000
Female	122 (41.1)	122 (41.1)		47 (59.5)	48 (60.8)	
Initial T stage						
T1-T2	48 (16.2)	40 (13.5)	.419	9 (11.4)	8 (10.1)	1.000
T3-T4	249 (83.8)	257 (86.5)		70 (88.6)	71 (89.9)	
Initial N stage						
N0-N1	178 (59.9)	192 (64.6)	.271	42 (53.2)	43 (54.4)	1.000
N2	119 (40.1)	105 (35.4)		37 (46.8)	36 (45.6)	
Initial M stage						
M0	142 (47.8)	138 (46.5)	.805	36 (45.6)	38 (48.1)	.873
M1	155 (52.2)	159 (53.5)		43 (54.4)	41 (51.9)	
Initial stage						
I-II	58 (19.5)	54 (18.2)	.753	11 (13.9)	9 (11.4)	.811
III-IV	239 (80.5)	243 (81.8)		68 (86.1)	70 (88.6)	
Previous colectomy						
No	142 (47.8)	143 (48.1)	1.000	6 (7.6)	7 (8.9)	1.000
Yes	155 (52.2)	154 (51.9)		73 (92.4)	72 (91.1)	
All RAS status						
Mutant	147 (49.5)	140 (47.1)	.622	61 (77.2)	52 (65.8)	.158
Wild	150 (50.5)	157 (52.9)		18 (22.8)	27 (34.2)	
BRAF status			1.000			1.000
Mutant	14 (4.7)	14 (4.7)		6 (7.6)	6 (7.6)	
Wild	283 (95.3)	283 (95.3)		73 (92.4)	73 (92.4)	
MMR status			1.000			1.000
Proficiency	276 (92.9)	276 (92.9)		71 (89.9)	71 (89.9)	
Deficiency	21 (7.1)	21 (7.1)		8 (10.1)	8 (10.1)	
Hypertension						
No	124 (41.8)	134 (45.1)	.456	42 (53.2)	31 (39.2)	.111
Yes	173 (58.2)	163 (54.9)		37 (46.8)	48 (60.8)	
Diabetic mellitus						
No	190 (64.0)	203 (68.4)	.298	55 (69.6)	48 (60.8)	.316
Yes	107 (36.0)	94 (31.6)		24 (30.4)	31 (39.2)	
Chronic kidney disease						
No	271 (91.2)	266 (89.6)	.577	73 (92.4)	72 (91.1)	1.000
Yes	26 (8.8)	31 (10.4)		6 (7.6)	7 (8.9)	

Abbreviations: MMR, mismatch repair gene; PSM, propensity score match.

**Table 3.** Cox regression analysis associated with overall survival in chemotherapy refractory patients with mCRC.

	Univariate	*P* value	Multivariate	*P* value
	HR		HR	
Treatment, TAS-102 vs Regorafenib	0.805 (0.678-0.957)	.0137	0.808 (0.679-0.961)	.0160
Age, ≦ 60 vs > 60	0.912 (0.784-1.062)	.2352	0.945 (0.806-1.109)	.4906
Gender, male vs female	0.827 (0.713-0.960)	.0123	0.828 (0.710-0.966)	.0163
Sideness, left side vs right side	0.753 (0.621-0.913)	.0040	0.741 (0.596-0.920)	.0067
Initial T stage, T1-T2 vs T3-T4	0.866 (0.680-1.103)	.2442	0.932 (0.710-1.223)	.6095
Initial N stage, N0-N1 vs N2	0.869 (0.734-1.029)	.1041	0.926 (0.769-1.115)	.4177
Initial M Stage, M0 vs M1	0.833 (0.704-0.985)	.0326	0.887 (0.730-1.078)	.2279
Initial stage, I-II vs III-IV	0.813 (0.652-1.013)	.0652	0.981 (0.737-1.305)	.8931
Colon surgery, no vs yes	0.995 (0.857-1.155)	.9468	0.944 (0.802-1.110)	.4854
All RAS status, wild vs mutant	0.950 (0.818-1.102)	.4963	0.950 (0.815-1.106)	.5070
BRAF status, wild vs mutant	0.926 (0.827-1.132)	.3411	0.910 (0.695-1.141)	.4580
MMR status, proficiency vs deficiency	0.941 (0.785-1.124)	.4136	0.930 (0.798-1.139)	.5117
Diabetes, no vs yes	0.964 (0.825-1.127)	.6463	0.921 (0.777-1.092)	.3437
Hypertension, no vs yes	0.949 (0.817-1.102)	.4897	0.893 (0.751-1.061)	.1980
Chronic kidney disease, no vs yes	0.979 (0.766-1.251)	.8639	0.958 (0.743-1.235)	.7425

Abbreviations: mCRC, metastatic colorectal cancer; HR, hazard ratio.

**Figure 3.**
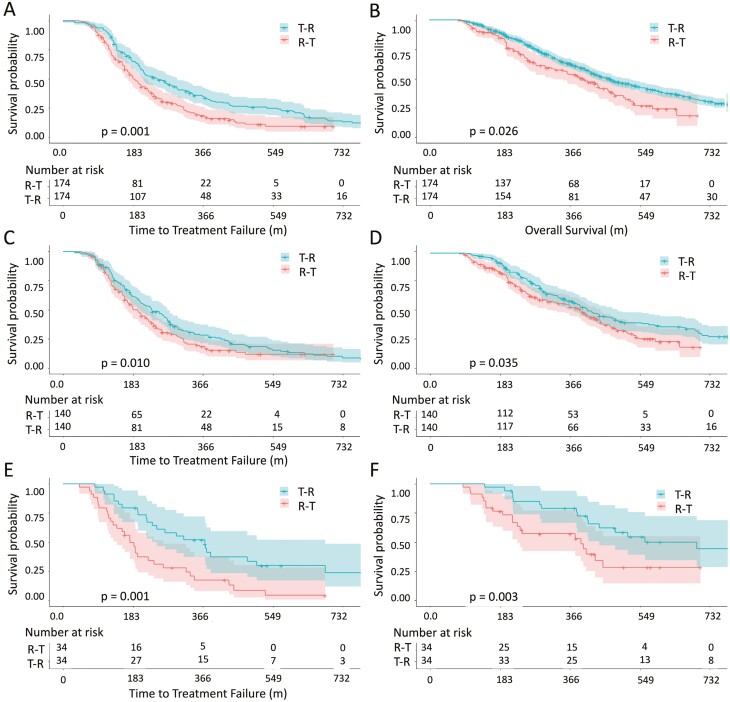
TTF and OS of metastatic colorectal cancer treated with TAS-102 followed by regorafenib (T-R) or regorafenib followed by TAS-102 (R-T). A and B, TTF and OS of total metastatic colorectal cancer treated with T-R or R-T. C and D, TTF and OS of metastatic left-side colorectal cancer treated with T-R or R-T. E and F, TTF and OS of metastatic right-side colorectal cancer treated with T-R or R-T.

Subgroup analysis regarding treatment sequences was investigated. Table 4 shows the distributions of characteristics between TAS-102 followed by regorafenib (T-R) and regorafenib followed by TAS-102 (R-T) groups. The median age was older in T-R when compared with those in R-T. Male was the predominant gender in both groups. In terms of sidedness, 80% had left-side colon cancer and approximately 20% had right-side colon cancer regardless T-R or R-T group. Apparently 85% of our patients was diagnosed to have clinical stages III and IV in both groups. Nearly 60% of patients had previous history of radical colectomy in both groups. As for all RAS status, 54% had RAS mutant while 46% had RAS wild type in our cohort. Similarly, there were 5% of our patients harboring BRAF mutant and 8% harboring dMMR. With regard to comorbidities, more patients with hypertension were found in R-T group while diabetic mellitus and chronic kidney disease were insignificant between both groups. After matching, 348 patients were analyzed for outcomes comparison, including 174 patients in T-R group and 174 patients in R-T group. The median ages were 62.6 years and male was the predominant gender (58%) in both groups. There were no significant differences in age, gender, sidedness, initial stage, all RAS status, BRAF status, MMR status, previous history of colectomy, diabetes, hypertension nor chronic kidney disease between T-R and R-T groups. Kaplan-Meier curves with TTF and OS were plotted for total population (Figure 3A and B). In total population, TTF and OS had significant differences between T-R and R-T groups. Median TTF and OS were 8.5 months versus 6.3 months (HR: 0.654, 95% CI: 0.513-0.833, *P* = .001) and 14.4 months versus 12.6 months (HR: 0.739, 95% CI: 0.557-0.981, *P* = .026) in T-R and R-T groups, respectively.

**Table 4.** Basic characteristics of chemotherapy refractory patients with mCRC, stratified by PSM.

	PSM unmatched			PSM matched		
	T-R	R-T	*P*	T-R	R-T	*P*
*N*	174	777		174	174	
Age (mean (SD))	62.67 (10.91)	61.55 (11.27)	.231	62.67 (10.91)	62.57 (10.73)	.929
Gender						
Male	100 (57.5)	415 (53.4)	.375	100 (57.5)	101 (58.0)	1.000
Female	74 (42.5)	362 (46.6)		74 (42.5)	73 (42.0)	
Primary tumor location						
Left	140 (80.5)	631 (81.2)	.904	140 (80.5)	136 (78.2)	.691
Right	34 (19.5)	146 (18.8)		34 (19.5)	38 (21.8)	
Initial T stage						
T1-T2	28 (16.1)	105 (13.5)	.444	28 (16.1)	17 (9.8)	.110
T3-T4	146 (83.9)	672 (86.5)		146 (83.9)	157 (90.2)	
Initial N stage						
N0-N1	101 (58.0)	478 (61.5)	.446	101 (58.0)	110 (63.2)	.380
N2	73 (42.0)	299 (38.5)		73 (42.0)	64 (36.8)	
Initial M stage						
M0	75 (43.1)	353 (45.4)	.636	75 (43.1)	85 (48.9)	.333
M1	99 (56.9)	424 (54.6)		99 (56.9)	89 (51.1)	
Initial stage						
I-II	27 (15.5)	113 (14.5)	.834	27 (15.5)	29 (16.7)	.884
III-IV	147 (84.5)	664 (85.5)		147 (84.5)	145 (83.3)	
Previous colectomy						
No	75 (43.1)	309 (39.8)	.468	75 (43.1)	70 (40.2)	.664
Yes	99 (56.9)	468 (60.2)		99 (56.9)	104 (59.8)	
All RAS status						
Mutant	96 (55.2)	412 (53.0)	.668	96 (55.2)	95 (54.6)	.793
Wild	78 (44.8)	365 (47.0)		78 (44.8)	79 (45.4)	
BRAF status			.985			1.000
Mutant	9 (5.2)	40 (5.1)		9 (5.2)	9 (5.2)	
Wild	165 (94.8)	737 (94.9)		165 (94.8)	165 (94.8)	
MMR status			.883			1.000
Proficiency	160 (92)	711 (91.5)		160 (92)	160 (92)	
Deficiency	14 (8.0)	66(8.5)		14 (8.0)	14 (8.0)	
Hypertension						
No	71 (40.8)	393 (50.6)	.025	71 (40.8)	87 (50.0)	.106
Yes	103 (59.2)	384 (49.4)		103 (59.2)	87 (50.0)	
Diabetic mellitus						
No	116 (66.7)	537 (69.1)	.590	116 (66.7)	112 (64.4)	.735
Yes	58 (33.3)	240 (30.9)		58 (33.3)	62 (35.6)	
Chronic kidney disease						
No	157 (90.2)	712 (91.6)	.655	157 (90.2)	154 (88.5)	.728
Yes	17 (9.8)	65 (8.4)		17 (9.8)	20 (11.5)	

Abbreviations: PSM, propensity score match; T-R, TAS-102 followed by regorafenib; R-T, regorafenib followed by TAS-102; MMR, mismatch repair gene.

Patients were stratified according to sidedness, accounting for 280 patients with left-side colon cancer and 68 patients with right-side colon. For patients with left-side colon cancer, 140 patients treated with T-R and 140 patients treated with R-T. For patients with right-side colon cancer, 34 patients treated with T-R and 34 patients treated with R-T. Table 5 shows the characteristics of patients in each group. The median age was 63 years in patients with left-side colon and the median age was 61 years in patients with right-side colon. There were no significant differences in age, gender, sidedness, initial stage, all RAS status, BRAF status, MMR status, previous history of colectomy, diabetes, hypertension nor chronic kidney disease between T-R and R-T groups, no matter for patients with left-side colon cancer or right-side colon cancer. Figure 3C and D plotted the TTF and OS of left-side colon group with Kaplan-Meier curves. For patients with left-side colon, median TTF and OS were 8.1 months versus 6.3 months (HR: 0.673, 95% CI: 0.545-0.831, *P* = .010) and 14.8 months versus 12.5 months (HR: 0.703, 95% CI: 0.584-0.901, *P* = .035) in T-R and R-T, respectively. Figure 3E and F plotted the TTF and OS of right-side colon group with Kaplan-Meier curves. For patients with right-side colon, median TTF and OS were 11.8 months versus 6.3 months (HR: 0.427, 95% CI: 0.245-0.743, *P* = .001) and 18.3 months versus 13.0 months (HR: 0.458, 95% CI: 0.234-0.818, *P* = .003) in T-R and R-T, respectively.

**Table 5.** Basic characteristics of chemotherapy refractory patients with mCRC, stratified by sideness.

	Left side			Right side		
	T-R	R-T	*P*	T-R	R-T	*P*
*N*	140	140		34	34	
Age (mean (SD))	63.04 (11.17)	62.89 (10.94)	.910	61.15 (9.77)	61.00 (10.92)	.954
Gender						
Male	87 (62.1)	88 (62.9)	1.000	13 (38.2)	13 (38.2)	1.000
Female	53 (37.9)	52 (37.1)		21 (61.8)	21 (61.8)	
Initial T stage						
T1-T2	21 (15.0)	19 (13.6)	.864	7 (20.6)	7 (20.6)	1.000
T3-T4	119 (85.0)	121 (86.4)		27 (79.4)	27 (79.4)	
Initial N stage						
N0-N1	80 (57.1)	90 (64.3)	.271	21 (61.8)	18 (52.9)	.624
N2	60 (42.9)	50 (35.7)		13 (38.2)	16 (47.1)	
Initial M stage						
M0	58 (41.4)	61 (43.6)	.809	17 (50.0)	20 (58.8)	.626
M1	82 (58.6)	79 (56.4)		17 (50.0)	14 (41.2)	
Initial stage						
I-II	21 (15.0)	13 (9.3)	.200	6 (17.6)	6 (17.6)	1.000
III-IV	119 (85.0)	127 (90.7)		28 (82.4)	28 (82.4)	
Previous colectomy						
No	72 (51.4)	70 (50.0)	.905	3 (8.8)	3 (8.8)	1.000
Yes	68 (48.6)	70 (50.0)		31 (91.2)	31 (91.2)	
All RAS status						
Mutant	68 (48.6)	70 (50.0)	.905	28 (82.4)	21 (61.8)	.105
Wild	72 (51.4)	70 (50.0)		6 (17.6)	13 (38.2)	
BRAF status			1.000			1.000
Mutant	6 (4.3)	6 (4.3)		3 (8.8)	3 (8.8)	
Wild	134 (95.7)	134 (95.7)		31 (91.2)	31 (91.2)	
MMR status			1.000			1.000
Proficiency	130 (92.9)	130 (92.9)		30 (88.2)	30 (88.2)	
Deficiency	10 (7.1)	10 (7.1)		4 (11.8)	4 (11.8)	
Hypertension						
No	55 (39.3)	76 (54.3)	.017	16 (47.1)	19 (55.9)	.627
Yes	85 (60.7)	64 (45.7)		18 (52.9)	15 (44.1)	
Diabetic mellitus						
No	94 (67.1)	97 (69.3)	.797	22 (64.7)	18 (52.9)	.460
Yes	46 (32.9)	43 (30.7)		12 (35.3)	16 (47.1)	
Chronic kidney disease						
No	127 (90.7)	128 (91.4)	1.000	30 (88.2)	31 (91.2)	1.000
Yes	13 (9.3)	12 (8.6)		4 (11.8)	3 (8.8)	

Abbreviations: PSM, propensity score match; T-R, TAS-102 followed by regorafenib; R-T, regorafenib followed by TAS-102; MMR, mismatch repair gene.

## Discussion

Our study has the largest cohort investigating the association between sidedness and survival among patients with chemo refractory mCRC treated with TAS-102 and regorafenib. Our study demonstrated that TTF was insignificant between TAS-102 and regorafenib, while OS was significant longer in TAS-102 group than in regorafenib group regardless the primary tumor location. Subgroup analysis of treatment sequence showed that TTF and OS were significant better in T-R group than in R-T group in total population. Survival benefits were more prominent in right-side colon than in left-side colon patients. After multivariate analysis, TAS-102 and left-side colon cancer were strongly related to OS. Our study was a retrospective nationwide population-based study with propensity-score matching analysis. Further prospective studies with randomized control were warranted to confirm with our results.

Both TAS-102 and regorafenib gain the indication of refractory mCRC.^19^ The pivotal phase III RECOURSE trial compared TAS-102 with placebo in patients with refractory mCRC and demonstrated that TAS-102 significantly prolonged OS (7.1 months vs 5.3 months, *P* < .001) and progression-free survival (PFS) (2.0 months vs. 1.7 months, *P* < .001) as compared with placebo.^18^ The pivotal phase III CORRECT trial compared regorafenib and placebo in patients with refractory mCRC and demonstrated that regorafenib resulted in significantly longer OS (6.4 months vs. 5.0 months, *P*: .0052) and PFS (1.9 months vs. 1.7 months, *P* < .0001) as compared with placebo. Based on these results, the PFS was 1.9-2 months and OS was 6-7 months in TAS-102 and regorafenib. Several retrospective studies compared the oncologic outcomes between TAS-102 and regorafenib with variable results.^20-22^ Thus, Su et al performed meta-analysis to compare the efficacy and toxicity of regorafenib and TAS-102,^24^ which showed survival did not significantly differ between these groups. Furthermore, a subgroup analysis of REGOTAS showed the OS was comparable between the regorafenib and TAS-102 groups regardless of primary tumor location.^25^ Our nationwide population-based study found that TTF data were consistent with previous literatures, but OS data differ from preceding studies. The reason why our OS was different between TAS-102 and regorafenib were unclear. One possible explanation was that patients treated with TAS-102 in our study seemed to have more subsequent treatments leading to longer OS. According to our study, 68% of TAS-102 patients had subsequent treatments with 46% regorafenib and 22% chemotherapy re-challenge, while 52% of regorafenib patients had subsequent treatments with 40% TAS-102 and 12% chemotherapy re-challenge. A latest phase III SUNLIGHT study demonstrated that TAS-102 plus bevacizumab resulted in longer OS than TAS-102 monotherapy in patients with chemotherapy refractory mCRC.^26^ This study provided an updated standard practice pattern of TAS-102 in the treatment of mCRC. Taken together, we can reasonable infer that our findings would hold true for TAS-102 plus bevacizumab.

As for treatment sequences, there are limited data focus on treatment sequence. A retrospective study collecting 49 patients with chemotherapy refractory colorectal cancer found median OS were insignificant difference between these 2 sequences and both sequences, T-R and R-T, can extend survival benefits.^27^ Another real world evidence showed in crossover use of TAS-102 and regorafenib, both sequences showed similar efficacy in terms of OS.^28^ A more recent prospective study in French presented that only 24% of their patients received R-T or T-R sequence with similar median OS from first treatment.^29^ A subgroup analysis from REGOTAS evaluating treatment sequence also demonstrated that the median OS values of R-T and T-R were also insignificant, accounting for 10.5 months and 9.4 months, respectively (*P* = .52).^30^ However, these patient numbers were very small in abovementioned studies. Recently, a retrospective study conducted at 13 Italian cancer institutes with a larger cohort of 146 patients in R-T group and 116 patients in T-R group firstly reported the median OS is significantly longer in the R-T group than in the T-R group (15.9 months versus 13.9 months, *P* = .0194).^31^ Our study had the largest population and showed TTF and OS were significantly longer toward T-R group than R-T group. To compare with Italian study, our study had less RAS mutation and more left-side colorectal cancer patients. This suggested our study included more chemotherapy-sensitive patients and displayed better responses to chemotherapy. Different population might lead to different results. Further studies focusing on the relationship between RAS status and survival in metastatic colorectal cancer treated with TAS-102 and regorafenib are warranted to answer these issues.

There are several potential limitations in our work, which are inherent to any retrospective studies. Chemotherapy regimen was decided at the discretion of physicians and patients, rather than randomized control. This might be the major bias in this study. Since regorafenib was reimbursement earlier than TAS-102, more patients in 2015-2017 were treated with regorafenib and more patients in 2018-2020 were treated with TAS-102. This will also have selection biases. However, in order to diminish this selection bias, patients in TAS-102 versus regorafenib and T-R versus R-T were all matched by propensity score matching with year of treatment as matching factors. Furthermore, the dosing schedules of regorafenib, escalation or de-escalation, were not available in this retrospective study. Previous study had demonstrated that different dosing strategies will result in different outcomes.^32^ Our study did not take into consideration about the dosing information, which might influent our results. Meanwhile, variable subsequent treatment, heterogeneity of our patients and inconsistent follow-up interval may also limit the power of our study. Given that, our study firstly investigated the association between sidedness and survival in chemotherapy refractory patients with mCRC treated with TAS-102 or regorafenib. Our study confirmed again the importance of sidedness in treatment of patients with mCRC.

In conclusion, our study has the largest cohort investigating the association between sidedness and survival in chemotherapy refractory metastatic colorectal cancer patients treated with TAS-102, regorafenib, or both. After matching, our results indicated TAS-102 first, with or without regorafenib following, provided a better survival benefit in chemotherapy refractory patients with mCRC across all sidedness. Our study could be a real-world evidence for physicians who treat patients with mCRC. Further prospective randomized control studies are warranted to confirm our conclusions.

## Data Availability

The data underlying this article will be shared on reasonable request to the corresponding author.
