# Correction to: Association between sidedness and survival among chemotherapy refractory metastatic colorectal cancer patients treated with trifluridine/tipiracil or regorafenib

**DOI:** 10.1093/oncolo/oyaf009

**Published:** 2025-02-25

This is a correction to: Kai-Yuan Hsiao, Hsin-Pao Chen, Kun-Ming Rau, Kuang-Wen Liu, Ben-Chang Shia, Wei-Shan Chang, Hao-Yun Liang, Meng-Che Hsieh, Association between sidedness and survival among chemotherapy refractory metastatic colorectal cancer patients treated with trifluridine/tipiracil or regorafenib, *The Oncologist*, Volume 29, Issue 12, December 2024, Pages e1669–e1679, https://doi.org/10.1093/oncolo/oyae235

In the originally published version of the manuscript, there was an error in text in the **Methods** section. Under heading **Data source**, in the last sentence, the IRB number should read: “[...](EMRP-110-167),[...]” instead of: “[...](EMRP-109-012),[...]”.

The emendation has been made to the article.

